# Translation and cross-cultural adaptation of a standardized international questionnaire on use of alternative and complementary medicine (I-CAM - Q) for Argentina

**DOI:** 10.1186/s12906-016-1074-4

**Published:** 2016-03-31

**Authors:** Santiago Esteban, Fernando Vázquez Peña, Sergio Terrasa

**Affiliations:** Family and Community Medicine Division, Hospital Italiano de Buenos Aires, Tte. J. D. Peron 4272, Buenos Aires, Argentina

**Keywords:** I-CAM-Q, Complementary medicine, Alternative medicine, Questionnaire

## Abstract

**Background:**

The widespread and growing use of alternative and complementary medicine (CAM) worldwide has been thoroughly described. In Argentina the limited information on the use of CAM has been reported between 40 and 55 %. However, the rate of use is extremely variable worldwide. For this purpose the international questionnaire on the use of complementary and alternative medicines (I-CAM - Q), was developed. The implementation of a translated and cross-culturally adapted version of the questionnaire would allow for a reliable and standardized evaluation of the rate of use of CAM in Argentina. It would be a great step towards improving what we know about the healing habits of our population.

**Methods:**

The forward and back-translation method was used. Four translators were involved. A committee was commissioned to reconcile the different versions. The process of cross-cultural adaptation was made by consulting 17 alternative and complementary medicine experts using the DELPHI method. The retrieved questionnaire was evaluated in 18 patients sampled by convenience (9 men, different educational and self-reported health levels). The interviews consisted of three parts: an initial demographics questionnaire; the administration of the I-CAM-Q and finally the cognitive interview, which included reviewing the questionnaire and reexamining questions that generated doubts during the interview. The comprehension of the questions was also evaluated. As a last step, using the information obtained from the interviews, the final version of the questionnaire was drafted.

**Results and conclusion:**

The questionnaire seems to have been accepted by most patients during the interviews. Conflictive elements that emerged did not seem to have an impact on its administration. The flexibility of the questionnaire allowed to add professionals and practices which contributed to a more accurate local adaptation. Further research should focus on assessing the questionnaire’s psychometric performance and validity, which so far has not been done.

**Electronic supplementary material:**

The online version of this article (doi:10.1186/s12906-016-1074-4) contains supplementary material, which is available to authorized users.

## Background

The growing and widespread use of complementary and alternative medicine (CAM) at the global level has been widely reported [1–12] especially for people who suffer from chronic health problems [10, 13, 14]. However, the rate of use in the various reports is extremely variable Such differences between studies may be due to the great heterogeneity that exists among the various measurement tools used, implying difficulties for comparisons. These differences between questionnaires lie mainly in the form of the questions and in the different definitions used for CAM [15–18].

These issues suggest that the use of a standardized questionnaire would facilitate the comparison among different populations. For this purpose the international questionnaire on the use of alternative medicines and complementary (I-CAM - Q) was developed in 2006 and later on published in 2009 [19]. Since its publication, the I-CAM-Q has been used as a tool for measuring the use of CAM in a limited number of studies, some of which involved its translation and cross-cultural adaptation into different languages and contexts in countries such as Saudi Arabia [20], Japan [21], England, Spain, Italy the Netherlands, Romania [22] and Germany [23]. It has been implemented to measure the rate of use of CAM in patients with diabetes [24], inflammatory bowel disease [25] and was administered in populations of different race, different levels of health education [26] both in urban and rural populations [21].

The questionnaire’s dissemination at the international level required local processes of translation and cross-cultural adaptation. Several authors reported different problems during this process. For example, Re et al. [23] attributed many of the difficulties some patients presented in comprehending the questions, to the scarce experience most people have with CAM. On the other hand, Eardley et al. [22] reported problems with the definitions of many practices and/or treatments, and also a high proportion of errors or missing data in the self-administered version.

As mentioned before, a Spanish version for Spain Eardley et al. [22] has been developed. Nevertheless, we believe that an Argentinian version is granted based on two fundamental facts: First, Argentinian Spanish differs from Spanish from Spain, particularly in the colloquial form, thus many of the expressions may not be understood especially among users with lower educational levels. Secondly, given the differences in how these practices are regulated and the fact that there is a strong native tradition in CAMs in Argentina, the already developed Spanish version might not be valid because it might not include certain modalities that are popular in our country, thus leaving out valuable information.

In Argentina, information on the use of CAM is limited. The reported prevalence ranges between 40 and 55 % [27–30]. However and as we expressed previously, data are not comparable with information from other countries and even between regions of Argentina, since the questionnaires differ significantly from each other. Thus, the development of a standardized tool would allow for a proper evaluation of our population’s healing habits. For this reason we decided to translate and cross-culturally adapt the I-CAM-Q to Argentinian Spanish.

## Methods

### Structure of the I-CAM-Q questionnaire

The I-CAM-Q questionnaire consists of four sections. The first section asks about recent visits to different CAMs treatment providers. The second inquires about CAM treatments received from a physician. The third inquires on the consumption of medicinal products based on herbs, vitamins, minerals or homeopathic medicines. Finally, in the fourth section, the questions are focused around self-help practices implemented by many people to improve their health. Also, in relation to each item of each section, frequency of use, main reason for using the last time and level of benefit received from its use are explored.

### Translation and cross-cultural adaptation process (Fig. 1)

**Fig. 1:**
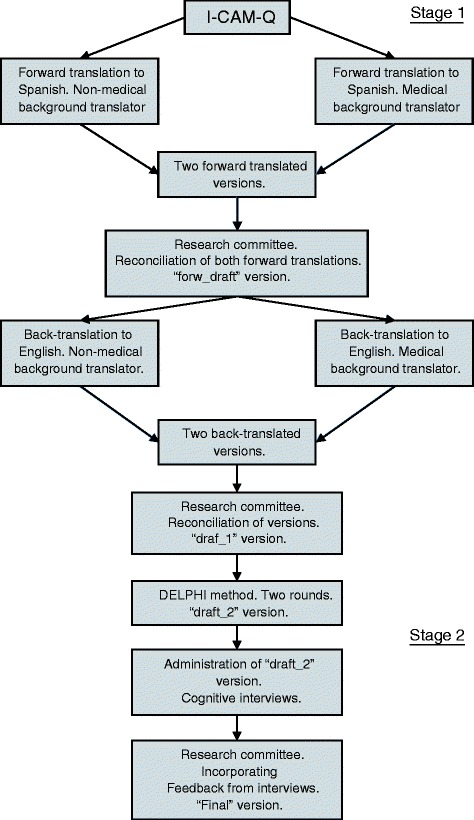
Overview of the translation and transcultural adaptation process

Initially the main author of the original version was contacted in order to obtain her authorization for the translation and cross-cultural adaptation of the questionnaire. Also aspects related to the form of administration were discussed. The author provided an interviewer administered version.

The process of translation and cross-cultural adaptation of the I-CAM - Q for use in Argentina had two stages. The first involved native English and Spanish translators and consultation with CAM experts, using the DELPHI method. The second stage involved the administration of the questionnaire and cognitive interviews to users of the health system in order to learn about their experience while responding it.

### First stage

As a *first step*, two independent translators, one with and one without medical background, performed a forward translation from English to Spanish. Both translators had Spanish as their mother tongue. Two versions of the questionnaire in Spanish were obtained. Both versions were evaluated by a committee consisting of both translators, the principal investigator and three researchers with a background in questionnaires. A preliminary version (“*forw_draft*”) was obtained by reconciling both forward translated versions.

In a *second step*, the “*forw*_*draft*” version was back-translated into English. It followed the same methodology as in step one, only both translators had English as their mother tongue. Two back-translated English versions were obtained (*back_draft 1 & 2*). The *third step* involved evaluating, comparing, and reconciling all versions *(original questionnaire, forw_draft, back_draft_1, back_draft_2)* into a single preliminary version “*draft_1”*. This was done by the same committee described before, this time including all four translators.

As a *fourth step* we sought to assess the cross-cultural aspects of the questionnaire. For this a DELPHI expert panel involving 17 experts was formed. The expert panel included physicians with expertise in CAM (family physicians and internal medicine specialists), pharmacists and non-physician CAM experts. Two rounds of questions were administrated via electronic questionnaires. The first round dealt basically with difficulties encountered by the Research Committee and previously published works. We sought a level of agreement equal or greater than 70 % in individual questions. The second round deepened on some questions of the first round, especially regarding those in which the 70 % agreement level was not reached. After this step, *“draft_2”* version was obtained.

### Second stage

Step number five involved the administration of version “*draft_2*” to 18 patients sampled by convenience in order to assess the questionnaire’s feasibility and applicability. Patients from the waiting area of a private medical center of the city of Buenos Aires (Hospital Italiano de Buenos Aires) and a free primary care clinic serving a low-income population (Centro de Salud San Pantaleón, Bajo Boulogne, San Isidro, Buenos Aires) were invited to join the study.

After giving oral consent, invited patients received an informational text that explained the objectives of the project and the confidential handling of their data.

The interviews were divided into three parts: 1) an initial demographics questionnaire; 2) the administration of the actual questionnaire *(“draft_2”)*, 3) Finally, the cognitive interview, which involved reviewing the questionnaire and revisiting those questions that generated doubts or were unclear to the participants in order to further assess the question’s comprehensibility. Based on previous experiences [22, 23] and after discussing it with the author of the questionnaire, we decided that, were the interviewees to inquire about a discipline or technique, then the interviewer should record it as a negative response.

The *sixth and final step* involved using the information obtained from the interviews and drafting a *“Final”* version of the I-CAM-Q translated and culturally adapted to Argentina.

### Ethics and consent

The research ethics board at Hospital Italiano de Buenos Aires (CEPI: Comité de Ética de protocolos de investigación del Hospital Italiano de Buenos Aires; *‘Ethics committee on research protocols at Hospital Italiano de Buenos Aires’*) approved the full protocol and determined that it was not necessary to obtain consent in writing; oral consent would suffice (approval process #2044).

### Data collection and data analysis

Unclear terms, expressions or words were recorded by the translators and later on discussed with the members of the Research Committee and the author of the original questionnaire via email.

Each individual’s responses were entered into an electronic version of the questionnaire. Cognitive interviews were recorded and then transcribed. Comments, suggestions, questions and responses that came up during the interviews were summarized and grouped into different categories.

## Results

### Research committee and expert group

During the translation process, discrepancies between the translators were addressed as well as the local interpretation of the different terms. This process was later on extended to the DELPHI group.

#### Treatment providers

We could not find a direct translation for *‘Treatment Provider’* since the term *‘Provider’* is not usually associated with health in our country. A feasible option was the Spanish word for *‘therapist’* but it is closely linked with psychotherapeutic treatments. Thus finally the term was translated as ‘*Professional or expert’.* It is worth noting that, initially, the research group had opted for the word *‘Professional’*, but during the DELPHI group rounds it did not reach the minimum agreement level, since many experts argued that it does not apply to all persons engaged in some of the therapeutic modalities considered in CAM, since in Argentina many of them do not have an enabling title or formal training.

‘*Herbalist’* has a straight forward translation as *‘Herbalista’*. However it is not a term used in Argentina. After the exchange with the group of experts, it was decided to use *‘Fitoterapeuta’*. Nonetheless it is not a very popular practice. It was therefore decided to add a brief description (“*an expert or professional who prescribes herbs, herbal teas, tinctures or medicinal plants*”).

There were no doubts about the translation of *‘Spiritual healer’ (‘Sanador Espiritual’)*. However, the expert group directly related the term to religious practices, which was not the description being sought by the authors of the questionnaire. It was decided to keep the term as is and revise it after the cognitive interviews.

The expert group suggested a list of terms often used in our country that could be included in the first column of the questionnaire: *Osteopathic expert, Reiki expert* and *Ayurvedic medicine expert. Also* ‘*Healer*’ *(Curandero) was proposed,* which is similar to a shaman and probably linked to a spiritual healer. The Group of experts was unable to reach a unanimous agreement on this. Many argued that a ‘*Healer’* could not be included as a *‘Professional or expert’* since it is not a uniform practice. It was concluded that it is a general denomination that includes various practices. Because of this it was decided to not include it in the list of suggested terms.

#### Treatments received from physicians (MDs)

In Argentina the term *‘Manipulation’* does not evoke the same meaning as in English. Therefore it was translated as *‘Joint manipulation and/or massage’ (Manipulación articular o masaje’).*

#### Self-Help practices

In Argentina *‘Self-Help’ (‘Autoayuda’)* is highly related to self-help books. Thus it was translated as *‘Personal practices that promote well-being’ (Prácticas personales que promueven el bienestar’). ‘Traditional healing ceremony’* was particularly difficult to translate since both the research team and the experts had no experience with this type of ceremonies. Based on previous experience from other research groups, it was decided to perform a direct translation *(‘Ceremonia de sanación tradicional’)* and assess its performance during the interviews.

*Meditation* as an isolated practice generated doubts among the experts about the possible overlap of this term with some other practices such as Yoga, Tai Ji Quan and Qi Gong.

#### Herbs and dietary supplements

Another item that needed an adapted translation was ‘*Herbs’*. It was replaced by ‘*Herbs, weeds and medicinal plants’ (‘Hierbas, yuyos y/o plantas medicinales).*

#### Reason for last use

A term that was greatly discussed within the group of experts was “*To improve well-being”*. Several experts raised the question if it was truly a third option or if it overlapped with some of the other two reasons for practice. Finally the conclusion was to test it in the interviews and evaluate the results.

In the options for the question “*What was the ****main ****reason you**** last**** saw a*, *was it* …?”, the term *Illness/Condition* was translated as a ‘*health problem’ (‘Problema de salud’)*, because it was considered to be more comprehensive, since many reasons for seeking CAM might not be seen as strictly illnesses or conditions by the interviewees.

#### Evaluation of the degree of perceived benefit

The question *“How helpful was it for you…?*” that is repeated in the last column of the four sections, was translated as ‘*How beneficial was it for you…?*’*(‘Cuán beneficioso le resultó…?’)*, since in Argentina, the word *‘useful’* has a very utilitarian denotation. This decision was made knowing that the meaning of the adjective “*beneficial*” does not have the same neutral dye that *‘useful’*.

### Interviews

Table 1 summarizes the socio-demographic characteristics of the subjects interviewed in order to assess the feasibility and applicability of the questionnaire.

**Table 1:** Socio-demographic characteristics of the respondents

Total	18
Women (%)	9 (50)
Age: Mean (SD)	46,5 (16,5)
- Men	44,4 (20)
- Women	48,8 (12,25)
Maximum level of education reached	(%)
Primary	22,2
Secondary	44,4
Tertiary	11,1
University	22,2
Self-reported health level	(%)
Excellent	5,6
Very good	33,3
Good	50
Fair	11,1
Poor	0
Reporting of at least one health problem	(%)
Yes	61
No	39

### Professionals and experts

The terms *physician, acupuncturist, homeopath or chiropractor* did not generate any conflicts among the responders. *‘Herbalist’* (*‘Fitoterapeuta’)* was a term for which it was decided to offer a description since in Argentina many different professionals or experts prescribe herbs or herbal teas. With the provided definition all of the responders were able to identify if they had seen such a professional or not.

As expected, *‘Spiritual healer’* was the most controversial item. Those interviewed associated the term basically with religion and cited examples of Evangelical and Catholic leaders. Only one of the patients related to ‘*spiritual healing’* with some aspects of Yoga: *- “would you include Yoga in this category?”.*

With respect to the term ‘*healers’ (‘Curanderos’)*, most responders related it mostly to non-healing practices giving it a negative connotation, so it was decided not to include it as one of the other types of treatment providers. For example, it was said that *“… they do not only dedicate themselves to heal, but mostly to do damage onto others…”*

### Herbs and dietary supplements

Most of the responders were unable to identify the differences between *herbs, vitamins/minerals and homeopathic remedies*. Furthermore, all persons who reported having taken some type of homeopathic medication had difficulty in adequately describing what they had consumed.

### Personal practices that promote well-being

In relation to ‘*meditation’*, a patient associated it with prayer, because many times in the Catholic religion ‘*meditation’ is referred* as a time of encounter with God: “…*meditation means to be silent and have an encounter with God…”.*

One of the responders asked if the term ‘*visualization’* referred to “… *visualize a problem of oneself (what is done with the help of a psychologist)”.* Many individuals hesitated when asked for a definition of this term and some mentioned *“to imagine landscapes or situations in order to be able to relax”.*

*‘To participate in a traditional healing ceremony’* generated some confusion among the responders. Everyone answered that they had not participated in such a ceremony in the last year, however, no one knew could give a clear definition of what they understood by it.

Five interviewees reported using ‘*relaxation techniques’*, but none reported having formally learnt any of them, but rather having just developed them intuitively, for example,”*sitting back and breathing deeply with your eyes closed trying to relax the body”.*

### Evaluation of the frequency of use

Questions in relation to the frequency of use of therapies in sections 1, 2 and 3 did not present any difficulties. However, the majority of respondents had difficulty trying to communicate the exact number of times that they had practiced some of the *‘Self-Help’* techniques during the past three months. They tended to describe it in the form of weekly or monthly frequency. When this occurred the interviewer assisted them in trying to define a number of times during the past three months, on the basis of the frequency that they communicated.

### Evaluation of reason for use

*‘To improve well-being’* was a very popular option to describe the reason for the use of any therapeutic modality. For example, many interviewees described this term as, *“… to be well in general, not only regarding a particular medical condition…”,“…it doesn’t cover only physical aspects, but also emotional ones…”*

### Evaluation of the degree of perceived benefit

Patients reported adequately distinguishing among the three levels of benefit: *very beneficial, somewhat beneficial or not at all beneficial.*

## Discussion

The final version of the questionnaire is provided in the Additional file 1. In order to improve the previously reported question routing problems associated with the self-administered version of the questionnaire [22, 23], we decided to use the interview format which also helps avoiding barriers such as illiteracy or blindness. Nevertheless, this could be a problem for future implementations involving larger numbers of participants.

A somewhat controversial issue, already discussed in the work of Re and Eardley [22, 23], was the possibility of including brief descriptions of either practices or treatment providers. For example, some authors decided to use versions which included definitions printed on the questionnaire, while others offered them to the interviewee upon request. We agreed with the author of the original version not to include any descriptions, assuming that if the interviewee did not recognize the term, it was most likely due to the fact that they had never come in contact with that particular practice or treatment provider. This decision may imply a decrease in the sensitivity of the question, but it probably improves the specificity by diminishing the proportion of false positives and thus, the probability of misclassification.

Although the majority of respondents reported not knowing many of the disciplines, after describing the practices to them, they confirmed not having used them. This seems to reinforce the notion that including descriptions might not be necessary.

Herbal medicine is a very common practice in Argentina. Some of the consulted experts raised the question that respondents might include many other treatments under Herbal medicine, which also include medicinal plants or natural extracts (*homeopathy, traditional Chinese medicine, healers*). However patients who were treated with homeopathic medicines were able to clearly distinguish between herbal medicine and homeopathy. This speaks of how clear the term *Homeopath/homeopathy* is defined for the general public. Similarly *physician, acupuncturist/acupuncture, chiropractic and joint manipulation* were adequately described by all participants. Furthermore, those who denied having consulted with any of these treatment providers were also able to provide a correct description of the practice, or, once the investigator offered them a definition, they confirmed their initial answer.

Both the expert group and the individuals interviewed linked *spiritual healing* with religious aspects. This direct connection is probably a local feature, since in Argentina, Christian religions are predominant, and there is not much dissemination of other types of spiritual development practices independently from religion. Eardley et al. [22] reported the same difficulty in various countries across Europe. We agreed with the author of the original version, that even though linking *‘spiritual healing’* to religion was not the original intent of this particular question, the answers should be considered valid, since that was what the person understood by *spiritual healing.*

Personal practices such as *Yoga, Tai Ji Quan and Qi Gong* are practices that include different types of meditation. However, participants distinguished them clearly and there was no overlap.

*‘To pray for your health’* was a widespread personal practice which was rated as ‘*very beneficial’ by all the participants*. With this in mind, more than half of the patients did not identify a specific reason for praying, but rather they reported doing it ‘*to improve their wellbeing.’*

The term ‘*traditional healing ceremony’* was the only one that could not be recognized or described by any of the participants. Even the experts failed to reach an agreement on what an adequate definition would be. Perhaps this term is clearer in communities with stronger aboriginal roots.

In agreement with Re et al. [23], we believe that the frequency of personal practices would be better reported by the respondents if it was expressed in terms of daily, weekly or monthly practice.

## Conclusion

The aim of this study was to obtain a translated and cross-culturally adapted version of the I-CAM – Q questionnaire. We believe that it was well accepted among the interviewed users, ratifying its flexibility in terms of adapting to local CAM habits. It is still a pending task to investigate the questionnaire’s psychometric performance and validity in our population.

## Additional file

Additional file 1:
**ICAMQ version for Argentina.** (DOCX 33 kb)

## Abbreviations

CAM: complementary and alternative medicine
I-CAM-Q: International Complementary and Alternative Medicine Questionnaire
