# Supplementary material for: Translation and cross-cultural adaptation of a standardized international questionnaire on use of alternative and complementary medicine (I-CAM - Q) for Argentina
Source: BMC Complement Altern Med. 2016 Mar 31;16:109. doi: 10.1186/s12906-016-1074-4 (PMC4815118; doi:10.1186/s12906-016-1074-4)
Supplement: Additional file 1: — ICAMQ version for Argentina. (DOCX 33 kb) [file 12906_2016_1074_MOESM1_ESM.docx]

***Cuestionario Internacional sobre Medicinas Alternativas y Complementarias (I-CAM-Q) – Versión para la Argentina***

***A. Consultas a profesionales o expertos.*** Los problemas de salud pueden ser tratados por diferentes profesionales. Quisiera hacerle algunas preguntas sobre esto.

| [PROFESIONAL o EXPERTO] | a. En los últimos 12 meses, consultó a un ________? | b. Cuántas veces consultó a un _____ en los últimos 3 meses? | c. Cuál fue el motivo *principal* por el que usted consultó a un ______ por *última vez*, fue por.....? | d. Cuán beneficioso le resultó ver a un _______? Diría que . . ? |
| --- | --- | --- | --- | --- |
| 1. Médico | **□** 1. Si (Preguntar A1b)  **□** 2. No (Pasar a A2)  **□** 8. No sabe (Pasar a A2)  **□** 9. No contesta (Pasar a A2) | **□** 00. Ninguna  **□** __ __ veces  **□** 77. No corresponde  **□** 88. No sabe  **□** 99. No contesta | **□** 1. Un problema de salud agudo, es decir, uno que duró menos de un mes?  **□** 2. Un problema de salud crónico, es decir, uno que duró más de un mes, ya sea para tratar el problema en sí o sus síntomas?  **□** 3. Para mejorar su bienestar  **□** 4. Otra, por favor especificar: ________________________________  **□** 7. No corresponde  **□** 8. No sabe  **□** 9. No contesta | **□** 1. Muy beneficioso  **□** 2. Algo beneficioso  **□** 3. Para nada beneficioso  **□** 7. No corresponde **□** 8. No sabe  **□** 9. No contesta |
| 2. Quiropráctico | **□** 1. Si (Preguntar A2b)  **□** 2. No (Pasar a A3)  **□** 8. No sabe (Pasar a A3)  **□** 9. No contesta (Pasar a A3) | **□** 00. Ninguna  **□** __ __ veces  **□** 77. No corresponde  **□** 88. No sabe  **□** 99. No contesta | **□** 1. Un problema de salud agudo, es decir, uno que duró menos de un mes?  **□** 2. Un problema de salud crónico, es decir, uno que duró más de un mes, ya sea para tratar el problema en sí o sus síntomas?  **□** 3. Para mejorar su bienestar  **□** 4. Otra, por favor especificar: ________________________________  **□** 7. No corresponde  **□** 8. No sabe  **□** 9. No contesta | **□** 1. Muy beneficioso  **□** 2. Algo beneficioso  **□** 3. Para nada beneficioso  **□** 7. No corresponde **□** 8. No sabe  **□** 9. No contesta |
| 3. Homeópata | **□** 1. Si (Preguntar A3b)  **□** 2. No (Pasar a A4)  **□** 8. No sabe (Pasar a A4)  **□** 9. No contesta (Pasar a A4) | **□** 00. Ninguna  **□** __ __ veces  **□** 77. No corresponde  **□** 88. No sabe  **□** 99. No contesta | **□** 1. Un problema de salud agudo, es decir, uno que duró menos de un mes?  **□** 2. Un problema de salud crónico, es decir, uno que duró más de un mes, ya sea para tratar el problema en sí o sus síntomas?  **□** 3. Para mejorar su bienestar  **□** 4. Otra, por favor especificar: ________________________________  **□** 7. No corresponde  **□** 8. No sabe  **□** 9. No contesta | **□** 1. Muy beneficioso  **□** 2. Algo beneficioso  **□** 3. Para nada beneficioso  **□** 7. No corresponde **□** 8. No sabe  **□** 9. No contesta |
| [PROFESIONAL O EXPERTO] | a. En los últimos 12 meses, consultó a un ________? | b. Cuántas veces consultó a un _____ en los últimos 3 meses? | c. Cuál fue el motivo *principal* por el que usted consultó a un ______ por *última vez*, fue por.....? | d. Cuán beneficioso le resultó ver a un _______? Diría que . . ? |
| 4. Acupunturista | **□** 1. Si (Preguntar A4b)  **□** 2. No (Pasar a A5)  **□** 8. No sabe (Pasar a A5)  **□** 9. No contesta (Pasar a A5) | **□** 00. Ninguna  **□** __ __ veces  **□** 77. No corresponde  **□** 88. No sabe  **□** 99. No contesta | **□** 1. Un problema de salud agudo, es decir, uno que duró menos de un mes?  **□** 2. Un problema de salud crónico, es decir, uno que duró más de un mes, ya sea para tratar el problema en sí o sus síntomas?  **□** 3. Para mejorar su bienestar  **□** 4. Otra, por favor especificar: ________________________________  **□** 7. No corresponde  **□** 8. No sabe  **□** 9. No contesta | **□** 1. Muy beneficioso  **□** 2. Algo beneficioso  **□** 3. Para nada beneficioso  **□** 7. No corresponde **□** 8. No sabe  **□** 9. No contesta |
| 5. Fitoterapeuta o Herbalista  (Un experto o profesional que receta yuyos, infusiones, tinturas y/o plantas medicinales incluyendo tratamientos con flores) | **□** 1. Si (Preguntar A5b)  **□** 2. No (Pasar a A6)  **□** 8. No sabe (Pasar a A6)  **□** 9. No contesta (Pasar a A6) | **□** 00. Ninguna  **□** __ __ veces  **□** 77. No corresponde  **□** 88. No sabe  **□** 99. No contesta | **□** 1. Un problema de salud agudo, es decir, uno que duró menos de un mes?  **□** 2. Un problema de salud crónico, es decir, uno que duró más de un mes, ya sea para tratar el problema en sí o sus síntomas?  **□** 3. Para mejorar su bienestar  **□** 4. Otra, por favor especificar: ________________________________  **□** 7. No corresponde  **□** 8. No sabe  **□** 9. No contesta | **□** 1. Muy beneficioso  **□** 2. Algo beneficioso  **□** 3. Para nada beneficioso  **□** 7. No corresponde **□** 8. No sabe  **□** 9. No contesta |
| 6. Sanador espiritual | **□** 1. Si (Preguntar A6b)  **□** 2. No (Pasar a A7)  **□** 8. No sabe (Pasar a A7)  **□** 9. No contesta (Pasar a A7) | **□** 00. Ninguna  **□** __ __ veces  **□** 77. No corresponde  **□** 88. No sabe  **□** 99. No contesta | **□** 1. Un problema de salud agudo, es decir, uno que duró menos de un mes?  **□** 2. Un problema de salud crónico, es decir, uno que duró más de un mes, ya sea para tratar el problema en sí o sus síntomas?  **□** 3. Para mejorar su bienestar  **□** 4. Otra, por favor especificar: ________________________________  **□** 7. No corresponde  **□** 8. No sabe  **□** 9. No contesta | **□** 1. Muy beneficioso  **□** 2. Algo beneficioso  **□** 3. Para nada beneficioso  **□** 7. No corresponde **□** 8. No sabe  **□** 9. No contesta |
| [PROFESIONAL o EXPERTO] | a. En los últimos 12 meses, consultó a un ________? | b. Cuántas veces consultó a un _____ en los últimos 3 meses? | c. Cuál fue el motivo *principal* por el que usted consultó a un ______ por *última vez*, fue por.....? | d. Cuán beneficioso le resultó ver a un _______? Diría que . . ? |
| 7. Ha visitado a algún otro profesional o experto en los últimos 12 meses?  Qué tipo de profesional ha visitado? _______________ | **□** 1. Si (Preguntar A7b)  **□** 2. No (Pasar a Sección B)  **□** 8. No sabe (Pasar a Sección B)  **□** 9. No contesta (Pasar a Sección B) | **□** 00. Ninguna  **□** __ __ veces  **□** 77. No corresponde  **□** 88. No sabe  **□** 99. No contesta | **□** 1. Un problema de salud agudo, es decir, uno que duró menos de un mes?  **□** 2. Un problema de salud crónico, es decir, uno que duró más de un mes, ya sea para tratar el problema en sí o sus síntomas?  **□** 3. Para mejorar su bienestar  **□** 4. Otra, por favor especificar: ________________________________  **□** 7. No corresponde  **□** 8. No sabe  **□** 9. No contesta | **□** 1. Muy beneficioso  **□** 2. Algo beneficioso  **□** 3. Para nada beneficioso  **□** 7. No corresponde **□** 8. No sabe  **□** 9. No contesta |

***B. Tratamientos realizados por Médicos.*** Quisiera preguntarle sobre tratamientos que haya recibido de parte de un médico en los últimos 12 meses.

[SI EL ENTREVISTADO NO HA VISITADO A UN MÉDICO EN LOS ÚLTIMOS 12 MESES, PASAR A LA SECCIÓN C]

| [TRATAMIENTO] | a. En los últimos 12 meses, ha recibido ______ de parte de un médico? | | b. Cuántas veces recibió _____ de parte de un médico en los últimos 3 meses? | c. Cuál fue el motivo *principal* por el que recibió ______ de parte de un médico por *última vez*? Fue ……? | d. Cuán beneficioso le resultó haber recibido _______ de parte de un médico? Diría que…..? |
| --- | --- | --- | --- | --- | --- |
| 1. Manipulación articular y/o masajes | **□** 1. Si (Preguntar B1b)  **□** 2. No (Pasar a B2)  **□** 8. No sabe (Pasar a B2)  **□** 9. No contesta (Pasar a B2) | | **□** 00. Ninguna  __ __ veces  **□** 77. No corresponde  **□** 88. No sabe  **□** 99. No contesta | **□** 1. Un problema de salud agudo, es decir, uno que duró menos de un mes?  **□** 2. Un problema de salud crónico, es decir, uno que duró más de un mes, ya sea para tratar el problema en sí o sus síntomas?  **□** 3. Para mejorar su bienestar  **□** 4. Otra, por favor especificar: ________________________________  **□** 7. No corresponde  **□** 8. No sabe  **□** 9. No contesta | **□** 1. Muy beneficioso  **□** 2. Algo beneficioso  **□** 3. Para nada beneficioso  **□** 7. No corresponde **□** 8. No sabe  **□** 9. No contesta |
| 2. Homeopatía | **□** 1. Si (Preguntar B2b)  **□** 2. No (Pasar a B3)  **□** 8. No sabe (Pasar a B3)  **□** 9. No contesta (Pasar a B3) | | **□** 00. Ninguna  __ __ veces  **□** 77. No corresponde  **□** 88. No sabe  **□** 99. No contesta | **□** 1. Un problema de salud agudo, es decir, uno que duró menos de un mes?  **□** 2. Un problema de salud crónico, es decir, uno que duró más de un mes, ya sea para tratar el problema en sí o sus síntomas?  **□** 3. Para mejorar su bienestar  **□** 4. Otra, por favor especificar: ________________________________  **□** 7. No corresponde  **□** 8. No sabe  **□** 9. No contesta | **□** 1. Muy beneficioso  **□** 2. Algo beneficioso  **□** 3. Para nada beneficioso  **□** 7. No corresponde **□** 8. No sabe  **□** 9. No contesta |
| 3. Acupuntura | | **□** 1. Si (Preguntar B3b)  **□** 2. No (Pasar a B4)  **□** 8. No sabe (Pasar a B4)  **□** 9. No contesta (Pasar a B4) | **□** 00. Ninguna  __ __ veces  **□** 77. No corresponde  **□** 88. No sabe  **□** 99. No contesta | **□** 1. Un problema de salud agudo, es decir, uno que duró menos de un mes?  **□** 2. Un problema de salud crónico, es decir, uno que duró más de un mes, ya sea para tratar el problema en sí o sus síntomas?  **□** 3. Para mejorar su bienestar  **□** 4. Otra, por favor especificar: ________________________________  **□** 7. No corresponde  **□** 8. No sabe  **□** 9. No contesta | **□** 1. Muy beneficioso  **□** 2. Algo beneficioso  **□** 3. Para nada beneficioso  **□** 7. No corresponde **□** 8. No sabe  **□** 9. No contesta |
| [TRATAMIENTO] | | a. En los últimos 12 meses, ha recibido ______ de parte de un médico? | b. Cuántas veces recibió _____ de parte de un médico en los últimos 3 meses? | c. Cuál fue el motivo *principal* por el que recibió ______ de parte de un médico por *última vez*? Fue ……? | d. Cuán beneficioso le resultó haber recibido _______ de parte de un médico? Diría que…..? |
| 4. Hierbas, Yuyos, Infusiones o Tinturas | | **□** 1. Si (Preguntar B4b)  **□** 2. No (Pasar a B5)  **□** 8. No sabe (Pasar a B5)  **□** 9. No contesta (Pasar a B5) | **□** 00. Ninguna  __ __ veces  **□** 77. No corresponde  **□** 88. No sabe  **□** 99. No contesta | **□** 1. Un problema de salud agudo, es decir, uno que duró menos de un mes?  **□** 2. Un problema de salud crónico, es decir, uno que duró más de un mes, ya sea para tratar el problema en sí o sus síntomas?  **□** 3. Para mejorar su bienestar  **□** 4. Otra, por favor especificar: ________________________________  **□** 7. No corresponde  **□** 8. No sabe  **□** 9. No contesta | **□** 1. Muy beneficioso  **□** 2. Algo beneficioso  **□** 3. Para nada beneficioso  **□** 7. No corresponde **□** 8. No sabe  **□** 9. No contesta |
| 5. Sanación espiritual | | **□** 1. Si (Preguntar B5b)  **□** 2. No (Pasar a B6)  **□** 8. No sabe (Pasar a B6)  **□** 9. No contesta (Pasar a B6) | **□** 00. Ninguna  __ __ veces  **□** 77. No corresponde  **□** 88. No sabe  **□** 99. No contesta | **□** 1. Un problema de salud agudo, es decir, uno que duró menos de un mes?  **□** 2. Un problema de salud crónico, es decir, uno que duró más de un mes, ya sea para tratar el problema en sí o sus síntomas?  **□** 3. Para mejorar su bienestar  **□** 4. Otra, por favor especificar: ________________________________  **□** 7. No corresponde  **□** 8. No sabe  **□** 9. No contesta | **□** 1. Muy beneficioso  **□** 2. Algo beneficioso  **□** 3. Para nada beneficioso  **□** 7. No corresponde **□** 8. No sabe  **□** 9. No contesta |
| 6. Ha recibido algún otro tipo de tratamiento de parte de un médico en los últimos 12 meses?  Qué tipo de tratamiento ha recibido? _______________ | | **□** 1. Si (Preguntar B6b)  **□** 2. No (Pasar a Sección C)  **□** 8. No sabe (Pasar a Sección C)  **□** 9. No contesta (Pasar a Sección C) | **□** 00. Ninguna  __ __ veces  **□** 77. No corresponde  **□** 88. No sabe  **□** 99. No contesta | **□** 1. Un problema de salud agudo, es decir, uno que duró menos de un mes?  **□** 2. Un problema de salud crónico, es decir, uno que duró más de un mes, ya sea para tratar el problema en sí o sus síntomas?  **□** 3. Para mejorar su bienestar  **□** 4. Otra, por favor especificar: ________________________________  **□** 7. No corresponde  **□** 8. No sabe  **□** 9. No contesta | **□** 1. Muy beneficioso  **□** 2. Algo beneficioso  **□** 3. Para nada beneficioso  **□** 7. No corresponde **□** 8. No sabe  **□** 9. No contesta |

***C. Uso de medicamentos a base de hierbas y suplementos dietarios.*** Además de los medicamentos que prescriben los médicos, algunas personas utilizan una gran variedad de otros productos para su salud, como por ejemplo, medicamentos a base de hierbas (yuyos) y suplementos dietéticos, que pueden ser comprimidos, cápsulas o líquidos (tés, tinturas, jarabes, etc.)

| C1. HIERBAS (YUYOS) | a. En los últimos 12 meses que hierbas ha utilizado? [Si ninguna, pasar a C2] | b. Utiliza actualmente ___________? | c. Cuál fue la principal razón por la que utilizó______ por última vez? Fue por . . .? | d. Cuán beneficioso le resultó _______?  Diría que . . . ? |
| --- | --- | --- | --- | --- |
| Hierba 1. ____________ | **□** 1. Si (Preguntar C1.1b)  **□** 2. No (Pasar a C1.2)  **□** 8. No sabe (Pasar a C1.2)  **□** 9. No contesta (Pasar a C1.2) | **□** 00. Ninguna  __ __ veces  **□** 77. No corresponde  **□** 88. No sabe  **□** 99. No contesta | **□** 1. Un problema de salud agudo, es decir, uno que duró menos de un mes?  **□** 2. Un problema de salud crónico, es decir, uno que duró más de un mes, ya sea para tratar el problema en sí o sus síntomas?  **□** 3. Para mejorar su bienestar  **□** 4. Otra, por favor especificar: ________________________________  **□** 7. No corresponde  **□** 8. No sabe  **□** 9. No contesta | **□** 1. Muy beneficioso  **□** 2. Algo beneficioso  **□** 3. Para nada beneficioso  **□** 7. No corresponde **□** 8. No sabe  **□** 9. No contesta |
| Hierba 2. ____________ | **□** 1. Si (Preguntar C1.2b)  **□** 2. No (Pasar a C2)  **□** 8. No sabe (Pasar a C2)  **□** 9. No contesta (Pasar a C2) | **□** 00. Ninguna  __ __ veces  **□** 77. No corresponde  **□** 88. No sabe  **□** 99. No contesta | **□** 1. Un problema de salud agudo, es decir, uno que duró menos de un mes?  **□** 2. Un problema de salud crónico, es decir, uno que duró más de un mes, ya sea para tratar el problema en sí o sus síntomas?  **□** 3. Para mejorar su bienestar  **□** 4. Otra, por favor especificar: ________________________________  **□** 7. No corresponde  **□** 8. No sabe  **□** 9. No contesta | **□** 1. Muy beneficioso  **□** 2. Algo beneficioso  **□** 3. Para nada beneficioso  **□** 7. No corresponde **□** 8. No sabe  **□** 9. No contesta |
| C2. VITAMINAS / MINERALES | a. En los últimos 12 meses, qué vitaminas o minerales ha utilizado?  [Si ninguna, pasar a C3] | b. Utiliza actualmente ___________? | c. Cuál fue la principal razón por la que utilizó______ por última vez? Fue por . . .? | d. Cuán beneficioso le resultó _______?  Diría que . . . ? |
| Vitamina/mineral 1. ____________ | **□** 1. Si (Preguntar C2.1b)  **□** 2. No (Pasar a C2.2)  **□** 8. No sabe (Pasar a C2.2)  **□** 9. No contesta (Pasar a C2.2) | **□** 00. Ninguna  __ __ veces  **□** 77. No corresponde  **□** 88. No sabe  **□** 99. No contesta | **□** 1. Un problema de salud agudo, es decir, uno que duró menos de un mes?  **□** 2. Un problema de salud crónico, es decir, uno que duró más de un mes, ya sea para tratar el problema en sí o sus síntomas?  **□** 3. Para mejorar su bienestar  **□** 4. Otra, por favor especificar: ________________________________  **□** 7. No corresponde  **□** 8. No sabe  **□** 9. No contesta | **□** 1. Muy beneficioso  **□** 2. Algo beneficioso  **□** 3. Para nada beneficioso  **□** 7. No corresponde **□** 8. No sabe  **□** 9. No contesta |
| Vitamina/mineral 2. ____________ | **□** 1. Si (Preguntar C2.2b)  **□** 2. No (Pasar a C3)  **□** 8. No sabe (Pasar a C3)  **□** 9. No contesta (Pasar a C3) | **□** 00. Ninguna  __ __ veces  **□** 77. No corresponde  **□** 88. No sabe  **□** 99. No contesta | **□** 1. Un problema de salud agudo, es decir, uno que duró menos de un mes?  **□** 2. Un problema de salud crónico, es decir, uno que duró más de un mes, ya sea para tratar el problema en sí o sus síntomas?  **□** 3. Para mejorar su bienestar  **□** 4. Otra, por favor especificar: ________________________________  **□** 7. No corresponde  **□** 8. No sabe  **□** 9. No contesta | **□** 1. Muy beneficioso  **□** 2. Algo beneficioso  **□** 3. Para nada beneficioso  **□** 7. No corresponde **□** 8. No sabe  **□** 9. No contesta |

| C3. REMEDIOS HOMEOPÁTICOS | a. En los últimos 12 meses, que remedios homeopáticos a utilizado?  [Si ninguno, finalice la entrevista] | b. Utiliza actualmente ___________? | c. Cuál fue la principal razón por la que utilizó______ por última vez? Fue por . . .? | d. Cuán beneficioso le resultó _______?  Diría que . . . ? |
| --- | --- | --- | --- | --- |
| Remedio homeopático  1. ____________ | **□** 1. Si (Preguntar C3.1b)  **□** 2. No (Pasar a sección D)  **□** 8. NO SABE (PASAR A C3.2)  **□** 9. NO CONTESTA (PASAR A C3.2) | **□** 00. Ninguna  __ __ veces  **□** 77. No corresponde  **□** 88. No sabe  **□** 99. No contesta | **□** 1. Un problema de salud agudo, es decir, uno que duró menos de un mes?  **□** 2. Un problema de salud crónico, es decir, uno que duró más de un mes, ya sea para tratar el problema en sí o sus síntomas?  **□** 3. Para mejorar su bienestar  **□** 4. Otra, por favor especificar: ________________________________  **□** 7. No corresponde  **□** 8. No sabe  **□** 9. No contesta | **□** 1. Muy beneficioso  **□** 2. Algo beneficioso  **□** 3. Para nada beneficioso  **□** 7. No corresponde **□** 8. No sabe  **□** 9. No contesta |
| Remedio homeopático  2. ____________ | **□** 1. Si (PREGUNTAR D3.2b)  **□** 2. No (Pasar a sección D)  **□** 8. NO SABE (Pasar a sección D)  **□** 9. NO CONTESTA (Pasar a sección D) | **□** 00. Ninguna  __ __ veces  **□** 77. No corresponde  **□** 88. No sabe  **□** 99. No contesta | **□** 1. Un problema de salud agudo, es decir, uno que duró menos de un mes?  **□** 2. Un problema de salud crónico, es decir, uno que duró más de un mes, ya sea para tratar el problema en sí o sus síntomas?  **□** 3. Para mejorar su bienestar  **□** 4. Otra, por favor especificar: ________________________________  **□** 7. No corresponde  **□** 8. No sabe  **□** 9. No contesta | **□** 1. Muy beneficioso  **□** 2. Algo beneficioso  **□** 3. Para nada beneficioso  **□** 7. No corresponde **□** 8. No sabe  **□** 9. No contesta |

***D. Prácticas personales que promueven el bienestar.*** Además de los medicamentos que prescriben los médicos, algunas personas suelen utilizar diferentes tipos de prácticas personales para mejorar su salud.

| [PRÁCTICAS PERSONALES] | a. En los últimos 12 meses, ha utilizado ________ como práctica personal? | b. Cuántas veces en los últimos 3 meses ha utilizado _______ como práctica personal? | c. Cuál fue la razón *principal* por la que utilizó _________ ? Fue….? | d. Cuán beneficioso le resultó _______?  Diría que . . . ? |
| --- | --- | --- | --- | --- |
| 1. Meditación | **□** 1. Si (Preguntar D1b)  **□** 2. No (Pasar a D2)  **□** 8. No sabe (Pasar a D2)  **□** 9. No contesta (Pasar a D2) | **□** 00. Ninguna  __ __ veces  **□** 77. No corresponde  **□** 88. No sabe  **□** 99. No contesta | **□** 1. Un problema de salud agudo, es decir, uno que duró menos de un mes?  **□** 2. Un problema de salud crónico, es decir, uno que duró más de un mes, ya sea para tratar el problema en sí o sus síntomas?  **□** 3. Para mejorar su bienestar  **□** 4. Otra, por favor especificar: _________________________  **□** 7. No corresponde  **□** 8. No sabe  **□** 9. No contesta | **□** 1. Muy beneficioso  **□** 2. Algo beneficioso  **□** 3. Para nada beneficioso  **□** 7. No corresponde **□** 8. No sabe  **□** 9. No contesta |
| 2. Yoga | **□** 1. Si (Preguntar D2b)  **□** 2. No (Pasar a D3)  **□** 8. No sabe (Pasar a D3)  **□** 9. No contesta (Pasar a D3) | **□** 00. Ninguna  __ __ veces  **□** 77. No corresponde  **□** 88. No sabe  **□** 99. No contesta | **□** 1. Un problema de salud agudo, es decir, uno que duró menos de un mes?  **□** 2. Un problema de salud crónico, es decir, uno que duró más de un mes, ya sea para tratar el problema en sí o sus síntomas?  **□** 3. Para mejorar su bienestar  **□** 4. Otra, por favor especificar: __________________________  **□** 7. No corresponde  **□** 8. No sabe  **□** 9. No contesta | **□** 1. Muy beneficioso  **□** 2. Algo beneficioso  **□** 3. Para nada beneficioso  **□** 7. No corresponde **□** 8. No sabe  **□** 9. No contesta |
| 3. Qi Gong (Chi Kung) | **□** 1. Si (Preguntar D3b)  **□** 2. No (Pasar a D4)  **□** 8. No sabe (Pasar a D4)  **□** 9. No contesta (Pasar a D4) | **□** 00. Ninguna  __ __ veces  **□** 77. No corresponde  **□** 88. No sabe  **□** 99. No contesta | **□** 1. Un problema de salud agudo, es decir, uno que duró menos de un mes?  **□** 2. Un problema de salud crónico, es decir, uno que duró más de un mes, ya sea para tratar el problema en sí o sus síntomas?  **□** 3. Para mejorar su bienestar  **□** 4. Otra, por favor especificar: __________________________  **□** 7. No corresponde  **□** 8. No sabe  **□** 9. No contesta | **□** 1. Muy beneficioso  **□** 2. Algo beneficioso  **□** 3. Para nada beneficioso  **□** 7. No corresponde **□** 8. No sabe  **□** 9. No contesta |
| [PRÁCTICAS PERSONALES] | a. En los últimos 12 meses, ha utilizado ________ como práctica personal? | b. Cuántas veces en los últimos 3 meses ha utilizado _______ como práctica personal? | c. Cuál fue la razón *principal* por la que utilizó _________ ? Fue….? | d. Cuán beneficioso le resultó _______?  Diría que . . . ? |
| 4. Tai Ji Quan (Tai Chi Chuan) | **□** 1. Si (Preguntar D4b)  **□** 2. No (Pasar a D5)  **□** 8. No sabe (Pasar a D5)  **□** 9. No contesta (Pasar a D5) | **□** 00. Ninguna  __ __ veces  **□** 77. No corresponde  **□** 88. No sabe  **□** 99. No contesta | **□** 1. Un problema de salud agudo, es decir, uno que duró menos de un mes?  **□** 2. Un problema de salud crónico, es decir, uno que duró más de un mes, ya sea para tratar el problema en sí o sus síntomas?  **□** 3. Para mejorar su bienestar  **□** 4. Otra, por favor especificar: ________________________________  **□** 7. No corresponde  **□** 8. No sabe  **□** 9. No contesta | **□** 1. Muy beneficioso  **□** 2. Algo beneficioso  **□** 3. Para nada beneficioso  **□** 7. No corresponde **□** 8. No sabe  **□** 9. No contesta |
| 5. Técnicas de relajación | **□** 1. Si (Preguntar D5b)  **□** 2. No (Pasar a D6)  **□** 8. No sabe (Pasar a D6)  **□** 9. No contesta (Pasar a D6) | **□** 00. Ninguna  __ __ veces  **□** 77. No corresponde  **□** 88. No sabe  **□** 99. No contesta | **□** 1. Un problema de salud agudo, es decir, uno que duró menos de un mes?  **□** 2. Un problema de salud crónico, es decir, uno que duró más de un mes, ya sea para tratar el problema en sí o sus síntomas?  **□** 3. Para mejorar su bienestar  **□** 4. Otra, por favor especificar: ________________________________  **□** 7. No corresponde  **□** 8. No sabe  **□** 9. No contesta | **□** 1. Muy beneficioso  **□** 2. Algo beneficioso  **□** 3. Para nada beneficioso  **□** 7. No corresponde **□** 8. No sabe  **□** 9. No contesta |
| 6. Visualización | **□** 1. Si (Preguntar D6b)  **□** 2. No (Pasar a D7)  **□** 8. No sabe (Pasar a D7)  **□** 9. No contesta (Pasar a D7) | **□** 00. Ninguna  __ __ veces  **□** 77. No corresponde  **□** 88. No sabe  **□** 99. No contesta | **□** 1. Un problema de salud agudo, es decir, uno que duró menos de un mes?  **□** 2. Un problema de salud crónico, es decir, uno que duró más de un mes, ya sea para tratar el problema en sí o sus síntomas?  **□** 3. Para mejorar su bienestar  **□** 4. Otra, por favor especificar: ________________________________  **□** 7. No corresponde  **□** 8. No sabe  **□** 9. No contesta | **□** 1. Muy beneficioso  **□** 2. Algo beneficioso  **□** 3. Para nada beneficioso  **□** 7. No corresponde **□** 8. No sabe  **□** 9. No contesta |
| [PRÁCTICAS PERSONALES] | a. En los últimos 12 meses, ha utilizado ________ como práctica personal? | b. Cuántas veces en los últimos 3 meses ha utilizado _______ como práctica personal? | c. Cuál fue la razón *principal* por la que utilizó _________ ? Fue….? | d. Cuán beneficioso le resultó _______?  Diría que . . . ? |
| 7. Participar de una ceremonia de sanación tradicional | **□** 1. Si (Preguntar D7b)  **□** 2. No (Pasar a D8)  **□** 8. No sabe (Pasar a D8)  **□** 9. No contesta (Pasar a D8) | **□** 00. Ninguna  __ __ veces  **□** 77. No corresponde  **□** 88. No sabe  **□** 99. No contesta | **□** 1. Un problema de salud agudo, es decir, uno que duró menos de un mes?  **□** 2. Un problema de salud crónico, es decir, uno que duró más de un mes, ya sea para tratar el problema en sí o sus síntomas?  **□** 3. Para mejorar su bienestar  **□** 4. Otra, por favor especificar: ________________________________  **□** 7. No corresponde  **□** 8. No sabe  **□** 9. No contesta | **□** 1. Muy beneficioso  **□** 2. Algo beneficioso  **□** 3. Para nada beneficioso  **□** 7. No corresponde **□** 8. No sabe  **□** 9. No contesta |
| 8. Rezar por su salud | **□** 1. Si (Preguntar D8b)  **□** 2. No (Pasar a D9)  **□** 8. No sabe (Pasar a D9)  **□** 9. No contesta (Pasar a D9) | **□** 00. Ninguna  __ __ veces  **□** 77. No corresponde  **□** 88. No sabe  **□** 99. No contesta | **□** 1. Un problema de salud agudo, es decir, uno que duró menos de un mes?  **□** 2. Un problema de salud crónico, es decir, uno que duró más de un mes, ya sea para tratar el problema en sí o sus síntomas?  **□** 3. Para mejorar su bienestar  **□** 4. Otra, por favor especificar: ________________________________  **□** 7. No corresponde  **□** 8. No sabe  **□** 9. No contesta | **□** 1. Muy beneficioso  **□** 2. Algo beneficioso  **□** 3. Para nada beneficioso  **□** 7. No corresponde **□** 8. No sabe  **□** 9. No contesta |
| 10. Utilizó alguna otra práctica personal (sin contar medicamentos a base de hierbas o suplementos dietéticos) en los últimos 12 meses?  Qué tipo de práctica personal utilizó? _______________ | **□** 1. Si (Preguntar D10b)  **□** 2. No (FINAL)  **□** 8. No sabe (FINAL)  **□** 9. No contesta (FINAL) | **□** 00. Ninguna  __ __ veces  **□** 77. No corresponde  **□** 88. No sabe  **□** 99. No contesta | **□** 1. Un problema de salud agudo, es decir, uno que duró menos de un mes?  **□** 2. Un problema de salud crónico, es decir, uno que duró más de un mes, ya sea para tratar el problema en sí o sus síntomas?  **□** 3. Para mejorar su bienestar  **□** 4. Otra, por favor especificar: ________________________________  **□** 7. No corresponde  **□** 8. No sabe  **□** 9. No contesta | **□** 1. Muy beneficioso  **□** 2. Algo beneficioso  **□** 3. Para nada beneficioso  **□** 7. No corresponde **□** 8. No sabe  **□** 9. No contesta |
